# Long‐Term Effects of Severe COVID‐19 on the Hippocampus: A 7T MRI Comparison with Alzheimer's Disease

**DOI:** 10.1002/alz70856_107515

**Published:** 2026-01-09

**Authors:** Beili Shao, Oluwatobi F Adeyemi, Penny Gowland, Richard Bowtell, Olivier Mougin, Mohammad Zia U.H Katshu, Elizabeta Mukaetova‐Ladinska, Monica Goss, Timothy D. Girard, Beth E. Snitz, Mary Ganguli, Heidi I.L. Jacobs, Farhaan S Vahidy, Tales Santini, Tamer S Ibrahim, Sudha Seshadri, Akram A. Hosseini

**Affiliations:** ^1^ School of Medicine, University of Nottingham, Nottingham, Nottinghamshire, United Kingdom; ^2^ Academic Neurology, Nottingham University Hospitals NHS Trust, Queen's Medical Centre, Nottingham, Nottinghamshire, United Kingdom; ^3^ Sir Peter Mansfield Imaging Centre, University of Nottingham, Nottingham, Nottinghamshire, United Kingdom; ^4^ School of Psychology, University of Leicester, Leicester, Leicestershire, United Kingdom; ^5^ Glenn Biggs Institute for Alzheimer's & Neurodegenerative Diseases, University of Texas Health Science Center, San Antonio, TX, USA; ^6^ University of Pittsburgh, Pittsburgh, PA, USA; ^7^ Massachusetts General Hospital, Boston, MA, USA; ^8^ Houston Methodist Research Institute, Houston, TX, USA; ^9^ TIRR Memorial Hermann, Houston, TX, USA

## Abstract

**Background:**

Post‐COVID cognitive dysfunction is a critical extrapulmonary complication of COVID‐19. A previous post‐mortem study has shown neuroinflammation and loss of hippocampal neurogenesis in COVID‐19 case. High‐resolution 7T MRI enables detailed assessment of hippocampal subfields, providing insights into disease process and early changes. This study explores hippocampal subfield volumes using 7T MRI in Alzheimer's disease (AD) and post‐COVID‐19 conditions, examining potential similarities in brain degeneration.

**Method:**

We analysed hippocampal subfield volumes in four groups: (1) AD patients with confirmed CSF‐Aβ status (*n* = 32), (2) individuals recovering from mild COVID‐19 >6 months post‐infection (Cv, *n* = 13), (3) individuals recovering from severe COVID‐19 with ICU admission >6 months prior (ICU‐Cv, *n* = 9), and (4) age‐matched healthy controls (HC, *n* = 29). Cognitive assessments, including the Montreal Cognitive Assessment (MOCA), were performed within these groups.

**Result:**

AD patients exhibited significant atrophy in multiple hippocampal subfields, including the entorhinal cortex (ERC), dentate gyrus (DG), hippocampal tail (Tail), and cornu ammonis (CA), compared to HC and Cv. In ICU‐Cv, ERC volume was significantly reduced compared to HC, while Cv showed no subfield differences from HC. AD patients had smaller ERC and DG volumes compared to ICU‐Cv. Average MOCA scores were lowest in AD (13.52), significantly lower than HC (27.58), Cv (27.24), and ICU‐Cv (24.78). ICU‐Cv showed a trend toward lower MOCA scores than HC, but differences were not significant. MOCA scores for ICU‐Cv were significantly lower than Cv. AD patients performed worse than ICU‐Cv on multiple cognitive domains, including verbal fluency (vegetable and animal naming), working memory (the longest digit span backward), visuospatial function (Benson figure copy), and episodic memory (total story recall and verbatim scoring). ICU‐Cv patients also scored lower on Benson figure copy (*p* = 0.048) and vegetable naming (*p* = 0.047) compared to HC.

**Conclusion:**

ERC, a key gateway for neocortical input to the hippocampus, is particularly susceptible in the early stages of AD, contributing to deficits in episodic memory and spatial navigation. In ICU‐Cv patients, we found selective atrophy of the ERC. This finding suggests that severe COVID‐19 may lead to targeted neurodegeneration through mechanisms distinct from AD but potentially overlapping in terms of hypoperfusion, inflammation, and metabolic stress.

